# The Story of the Insane from Year to Year

**Published:** 1904-05-21

**Authors:** 


### THE STORY OF THE INSANE FROM YEAR TO YEAR.

Sixteenth Annual Series.

### WEST SUSSEX ASYLUM, CHICHESTER.

This asylum contained 622 patients on the first of January and by the end of the year the number had risen to 728. There were 256 first admissions, 26 readmissions, 48 recoveries, 32 discharged relieved, 43 not improved, and 53 died. The recoveries are put down at 31·78 per cent. of the admissions, and the deaths at 7·58 per cent. of the average daily number resident. Of the deaths 23 were due to cerebral and spinal diseases, 2 to pneumonia, and 8 to consumption. Of the year's admissions the insanity was supposed to be caused by various moral causes in 23 instances, by intemperance in drink in 35, by hereditary influence in 97, previous attacks in 76, congenital defect in 17, and by influenza in 10. Many of the cases were in a state of chronic insanity when admitted, and therefore Dr. Kidd has but an indifferent supply of raw material to work on. He thinks recovery is probable in 50, possible in 56, improbable in 91, and impossible in 85. Lady Gordon Lennox evinces much interest in the care of the insane and during last year a bazaar was opened by her with the object of providing funds for rendering help to patients for which the ordinary funds are not available, presumably to help discharged cases. A sum of £80 was handed over to this asylum. Lectures continue to be given by the medical officers to the staff and 28 nurses and 15 attendants passed the First Aid examination lately held. We wish we could hear that Dr. Kidd had begun a three-years' course of training for his nurses, and so bring them up to the level of hospital nurses. A meeting of the asylum staff was held to discuss the question of pensions to asylum officials and the following resolution was passed:—"Pensions should be assured for those engaged in the care of the insane by a definite provision in the next Lunacy Bill, making superannuation allowances compulsory, and not as at present, discretionary." This leaves details untouched, and it was, perhaps, advisable not to load it in any way; at the same time, if absolute certainty is granted, and we are decidedly of opinion that it should be, it is almost certain to be on a lower scale than that which the existing Act permits but does not make claimable. However, the resolution was endorsed by the Visiting Committee and sent to the members of Parliament for the county. This is a step which, inaugurated by Dr. Kidd, we hope to see widely followed. The weekly cost of maintenance was 11s. 2¼d. per head.

### LANCASHIRE COUNTY ASYLUM AT RAINHILL.

At the beginning of the year there were in residence 2,103 patients, and on the last day of December the number had fallen to 2,079. There were 608 first admissions, 46 readmissions, 182 recoveries, 18 discharged relieved, 260 discharged not improved, and 218 died. The total number under care during the year was 2,747, and the average daily number resident was 2,061. The recoveries stand at 27·8[illegible] per cent. of the admissions, and the deaths at 10 57 per cent. of the average number resident. Of the year's deaths 7[illegible] were caused by cerebral and spinal diseases, 22 by pneumonia, bronchitis, and pleurisy, 52 by consumption, and 4 by colitis. Of the year's admissions 60 were owing to various moral causes. Drink heads the list among the physical causes with the enormous number of 243; heredity is responsible for 75, and previous attacks for 110. A new pathological department has been opened, and "is now placed on a thoroughly satisfactory basis." There is nothing special in Dr. Wiglesworth's report. The weekly charge per head is 9s. 11d.

